# Time-dependent prognostic value of coronary plaque burden and hyperemic myocardial blood flow

**DOI:** 10.1007/s00259-026-08135-3

**Published:** 2026-08-30

**Authors:** Jorge Dahdal, Teemu Maaniitty, Ruurt A. Jukema, Nick S. Nurmohamed, Pieter G. Raijmakers, Roel S. Driessen, Matias Mäenpää, Sarah Bär, R. Nils Planken, Niels van Royen, Jeroen J. Bax, Antti Saraste, Saloua el Messaoudi, Juhani Knuuti, Paul Knaapen, Ibrahim Danad

**Affiliations:** 1 Department of Cardiology, Amsterdam Cardiovascular Sciences, Amsterdam UMC, Vrije Universiteit Amsterdam, Amsterdam, the Netherlands; 2 Departamento de Enfermedades Cardiovasculares, Clínica Alemana Universidad del Desarrollo, Santiago, Chile; 3 Turku PET Centre, Turku University Hospital and University of Turku, Turku, Finland; 4 Department of Clinical Physiology, Nuclear Medicine and PET, Turku University Hospital, Turku, Finland; 5 Department of Radiology, St. Paul’s Hospital and University of British Columbia, Vancouver, BC Canada; 6 Department of Vascular Medicine, Amsterdam Cardiovascular Sciences, Amsterdam UMC, University of Amsterdam, Amsterdam, the Netherlands; 7 Radiology, Nuclear Medicine and PET Research, Amsterdam UMC, Vrije Universiteit Amsterdam, Amsterdam, the Netherlands; 8 Department of Cardiology, Bern University Hospital, Bern, Switzerland; 9 Department of Radiology, Mayo Clinic, Rochester, MN USA; 10 Department of Cardiology, Radboud University Medical Center, Geert Grooteplein Zuid 10, 6525 GA Nijmegen, the Netherlands; 11 Department of Cardiology, Leiden University Medical Center, Leiden, the Netherlands; 12 Heart Center, Turku University Hospital, Turku, Finland

**Keywords:** Coronary artery disease, Coronary computed tomography angiography, Positron emission tomography, Myocardial perfusion imaging, Quantitative plaque analysis, Risk stratification

## Abstract

**Rationale:**

The time-dependent contributions of coronary atherosclerotic plaque burden and myocardial ischemia to clinical outcomes in suspected coronary artery disease (CAD) remain uncertain. This study aimed to evaluate the temporal associations of quantitatively assessed plaque burden and ischemia with cardiovascular events.

**Methods:**

We studied 1,385 patients with suspected CAD who underwent coronary computed tomography angiography (CCTA) and [^15^O]H₂O positron emission tomography (PET) perfusion imaging. Plaque burden was measured as percent atheroma volume (PAV) using AI-based CCTA analysis. Myocardial perfusion was evaluated by regional hyperemic myocardial blood flow (hMBF) using PET. Adjusted Cox regression models, including both PAV and hMBF, were used to assess associations with a composite outcome (death, non-fatal myocardial infarction, unstable angina) at 3, 6, and 9 years.

**Results:**

The median follow-up time was 7.1 years, during which 185 patients (13.4%) experienced the outcome. Higher per-patient PAV (per 1%) was consistently and independently associated with increased event rate across all three timepoints (aHR 1.035–1.047, all *p* < 0.001). Lower regional hMBF (per 0.1 mL/min/g) was independently associated with events at 3 years (aHR 1.050, *p* = 0.004) and 6 years (aHR 1.028, *p* = 0.024), but not at 9 years (aHR 1.020, *p* = 0.063). After excluding early revascularization patients, regional hMBF remained associated with outcomes at all timepoints.

**Conclusions:**

Plaque burden and regional hMBF are independent markers of cardiovascular risk. Plaque burden remained consistently associated with adverse clinical outcomes throughout follow-up, whereas regional hMBF was primarily related to short- and intermediate-term events, likely influenced by the effects of myocardial revascularization.

**Graphical abstract:**

Cox models were developed incorporating both per-patient PAV and regional hMBF, with adjustment for clinical covariates and early revascularization status. AI = artificial intelligence; CCTA = coronary computed tomography angiography; PAV = percent atheroma volume; PET = positron emission tomography; hMBF = hyperemic myocardial blood flow; MI = myocardial infarction; UA = unstable angina.

**Figure:**
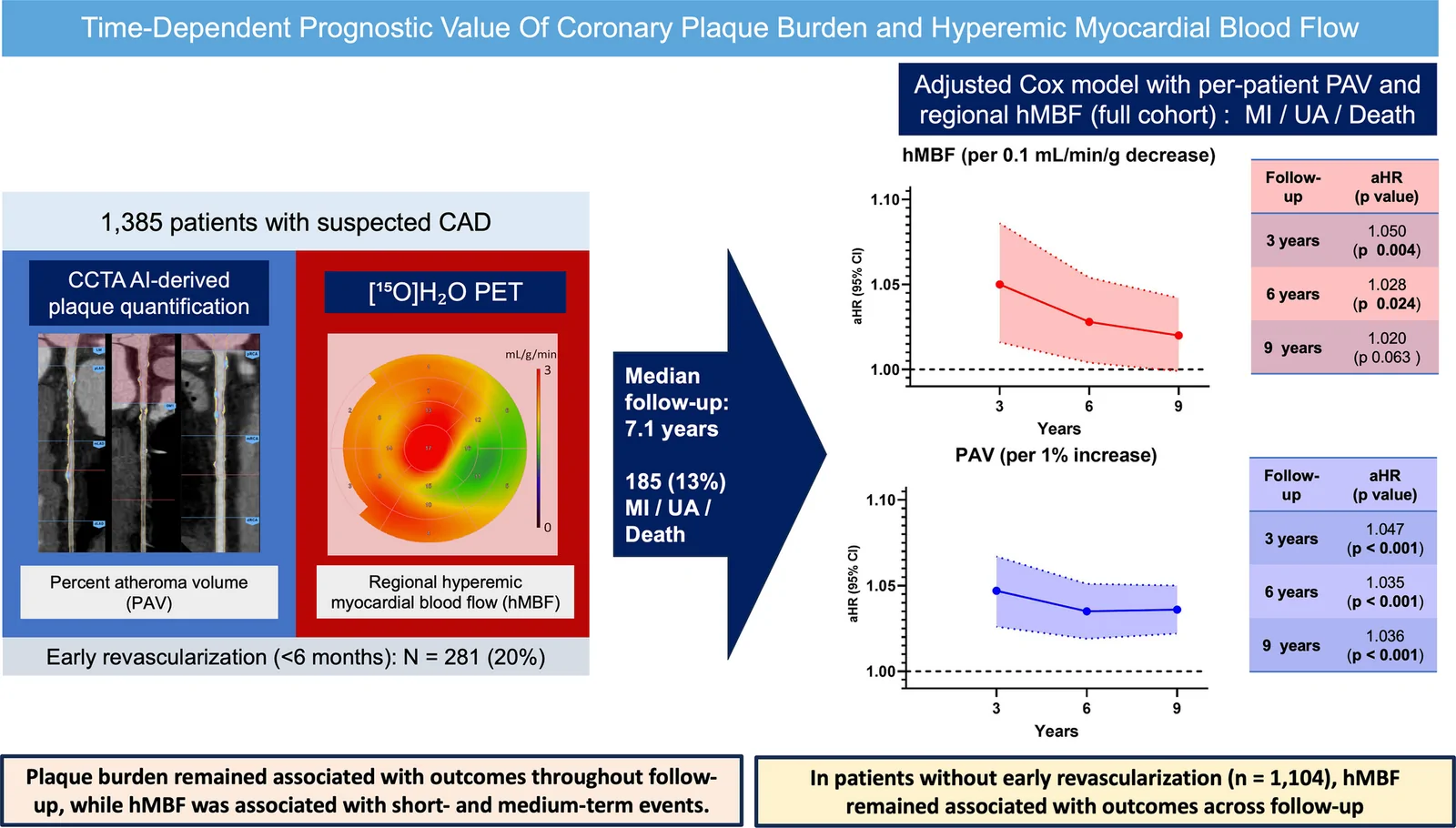

**Supplementary Information:**

The online version contains supplementary material available at https://doi.org/10.1007/s00259-026-08135-3.

## Introduction

Prognostic evaluation and clinical decision-making in coronary artery disease (CAD) have traditionally relied on the anatomical severity of coronary stenoses and the detection of associated myocardial ischemia. However, landmark trials in stable CAD have consistently shown that the anatomical extent of atherosclerosis predicts myocardial infarction (MI) and all-cause mortality more strongly and independently of ischemia [1, 2]. Nevertheless, large and well-conducted observational studies underscore that the extent and severity of myocardial ischemia are powerful predictors of cardiovascular outcomes, and that the benefit of revascularization might be modulated by the degree of ischemia [3, 4]. Additionally, emerging evidence suggests that coronary plaque burden derived from coronary computed tomography angiography (CCTA), now quantifiable using dedicated software powered by artificial intelligence (AI), is a stronger marker for risk stratification than conventional metrics of CAD extent [5–7].

While myocardial ischemia and coronary atherosclerotic burden are often interrelated, limited data exist on their independent prognostic value and how it changes over time. In this study, we assessed the time-dependent prognostic value of [^15^O]H₂O positron emission tomography (PET) -derived regional hyperemic myocardial blood flow (hMBF) and CCTA-derived AI-quantified coronary plaque burden.

## Methods

### Study cohort

Between 2007 and 2016, a total of 1,385 symptomatic patients with suspected chronic coronary syndrome and no prior history of CAD were included from two different centers: Turku University Hospital, Turku, Finland (*n* = 830, 60%) and Amsterdam University Medical Center, Amsterdam, the Netherlands (*n* = 555, 40%). All participants underwent CCTA and [^15^O]H₂O PET myocardial perfusion imaging (MPI). Imaging protocols varied between the institutions: at Turku University Hospital, PET MPI was selectively performed in patients with suspected obstructive CAD based on visual interpretation of CCTA, whereas in Amsterdam University Medical Center, combined CCTA and PET MPI were routinely conducted in all referred patients [8]. Subsequent patient management decisions, including revascularization and medical therapy, were made at the discretion of the treating physician, based on all available clinical and imaging data. The study was approved by the respective institutional ethics committees, which waived the requirement for written informed consent due to the retrospective nature of the study. All procedures were conducted in accordance with the Declaration of Helsinki.

### CCTA acquisition

CCTA was performed using ≥ 64-row scanners. Acquisition protocols have been described previously [8–10]. All patients received oral/sublingual nitrates before scanning. In addition, oral or intravenous beta-blockers were administered to lower heart rate below 65 beats per minute. CCTA was performed with prospective ECG gating using a tube voltage of 100–120 kVp and a tube current of 600–1,000 mA. Iodinated contrast material was injected according to institutional protocols.

### Coronary plaque quantification

**Fig. 1:**
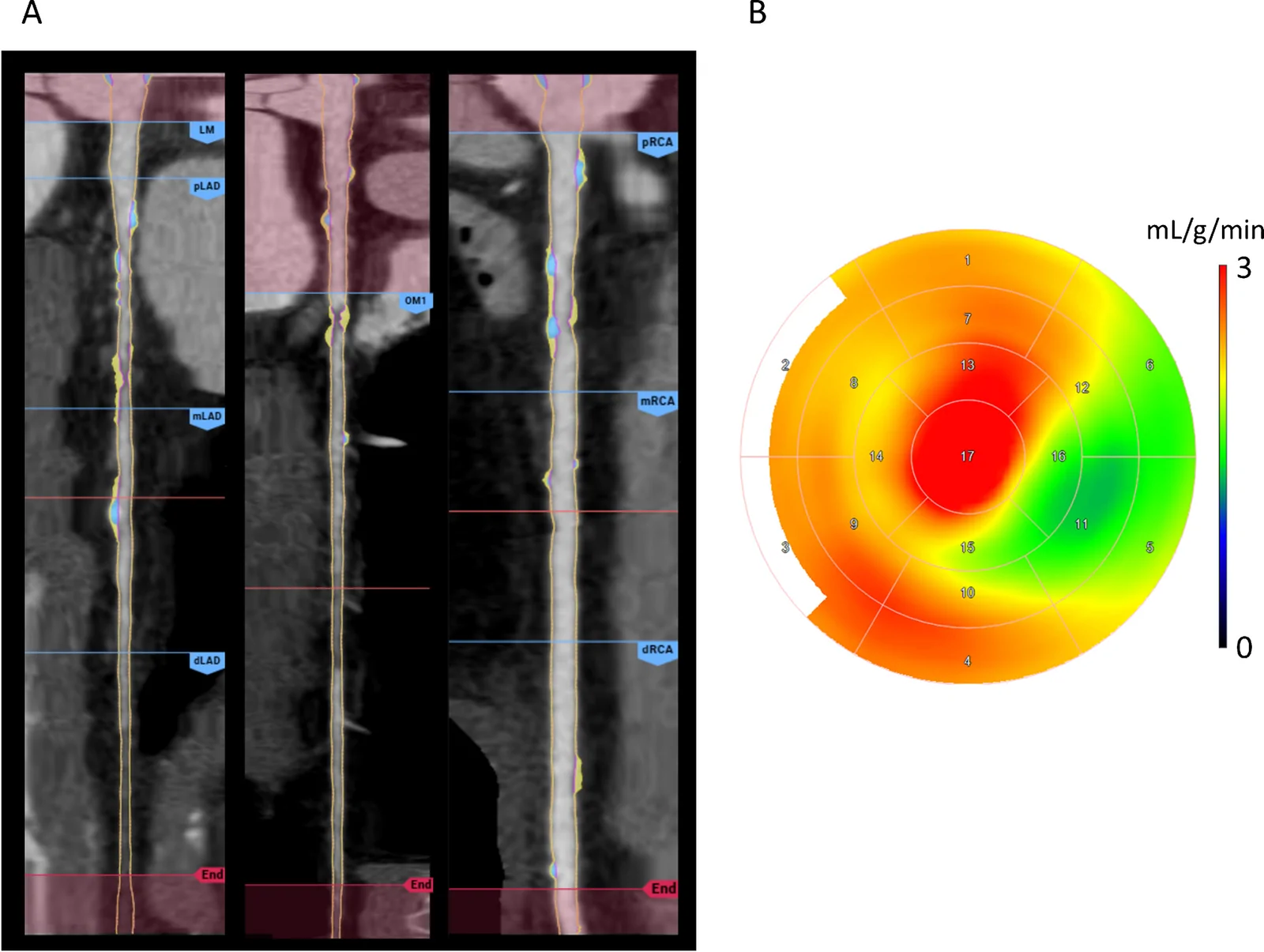
Case example. An example of quantitative AI-QCT analysis of CCTA and quantitative [^15^O]H₂O PET MPI in the same patient. **A** Quantitative AI-QCT plaque analysis showing non-calcified (yellow) and calcified (blue) atherosclerotic plaque in the LAD, in the OM branch of the LCx, and in the RCA. The TPV is 340 mm^3^, corresponding to a per-patient PAV of 10.9% (Stage 2 PAV) when normalized by the individual vessel volume of 3128 mm^3^. **B** hMBF by PET MPI is presented according to the standard 17-segment model, revealing a perfusion defect in the lateral left ventricular wall consistent with LCx ischemia (regional hMBF 1.7 mL/min/g, calculated as the lowest mean hMBF across two contiguous myocardial segments). Hyperemic perfusion is normal in the LAD and RCA territories. Global hMBF was 2.4 mL/min/g. CCTA = coronary computed tomography angiography; PET = positron emission tomography; MPI = myocardial perfusion imaging; LAD = left anterior descending artery; OM = obtuse marginal; LCx = left circumflex artery; RCA = right coronary artery; TPV = total plaque volume; PAV = percent atheroma volume; hMBF = hyperemic myocardial blood flow

CCTA images were analyzed using an FDA-cleared AI-software (AI-QCT, Cleerly Inc., Denver, Colorado, USA), as previously described (Fig. 1) [7, 11]. Anonymized datasets were transferred to a centralized core lab and analyzed blinded to clinical and outcome data. Coronary segments ≥ 1.5 mm in diameter were assessed, and coronary atherosclerosis was defined as any tissue structure ≥ 1 mm^2^ within the vessel wall, distinct from epicardial fat or lumen. Plaque volume (mm^3^) was quantified per lesion and summed to obtain total plaque volume (TPV) per patient. TPV was normalized to vessel volume and expressed as percent atheroma volume (PAV = TPV/vessel volume × 100%), as represented in Fig. 1. Segments with poor image quality were excluded by automated quality control; per-patient PAV remains a robust marker of total plaque burden despite such exclusions [12]. Per-patient PAV was analyzed primarily as a continuous variable and for categorical analyses, stratified into four stages: Stage 0 (no plaque), Stage 1 (> 0–5%), Stage 2 (> 5–15%), and Stage 3 (> 15%) [13]. PAV was also quantified at the vessel level for the left main and left anterior descending (LM–LAD), left circumflex (LCx), and right coronary artery (RCA), with left main plaque assigned to the LM–LAD territory. The maximum per-vessel PAV for each patient was defined as the highest of the three values. Percent diameter stenosis was quantified per lesion, and the maximum per-patient value was analyzed as a continuous variable.

### PET acquisition and image analysis

Myocardial perfusion PET scans were acquired with integrated PET/CT scanners, employing [^15^O]H₂O as a perfusion tracer. Patients were instructed to abstain from caffeine and xanthine-containing products for at least 24 h prior to the examination. Pharmacologic hyperemia was induced using intravenous adenosine at a rate of 140 μg/kg/min.

Quantitative myocardial blood flow (MBF) was assessed using in-house developed software packages, CardiacVUer (Amsterdam UMC, location VU Medical Center, Amsterdam, the Netherlands) and Carimas (Turku PET Centre, Turku, Finland), which generate parametric hMBF images (mL/min/g) across the standard 17-segment left ventricular model from the American Heart Association guidelines (Fig. 1) [14, 15]. A previous study demonstrated a high inter-software agreement for both global and regional hMBF measurements [16]. Regional hMBF, analyzed as a continuous variable, was defined at the patient level as the lowest mean hMBF across two contiguous segments; global hMBF was the mean hyperemic flow across all 17 segments. Regional hMBF from [^15^O]H₂O PET was chosen as the primary perfusion metric, being one of the parameters most widely used clinically to detect ischemia and guide revascularization. Unlike a whole-ventricle average, it is not diluted by normal myocardium and therefore captures focal as well as diffuse flow reduction, while retaining prognostic value comparable to global hMBF [17]. For categorical analyses, regional perfusion was classified as normal (hMBF > 2.3 mL/min/g) or impaired (≤ 2.3 mL/min/g) using a previously validated threshold [18]. Impaired perfusion was further graded as mildly (> 2.0–2.3 mL/min/g), moderately (> 1.5–2.0 mL/min/g), or severely reduced (≤ 1.5 mL/min/g).

### Follow-up

Clinical follow-up was conducted through a standardized process involving electronic medical records, telephone follow-up, and interrogation of national health registries. The outcome was a composite of all-cause mortality, non-fatal MI, and unstable angina (UA), and all identified events were verified by the study investigators according to the criteria outlined in the European Society of Cardiology guidelines [19]. Early revascularization was defined as PCI or CABG performed within 6 months of the index CCTA, consistent with prior work in this cohort [20, 21]. Events occurring in the context of early revascularization procedures were not included in the outcome analysis.

### Statistical analysis

Continuous variables were presented as mean ± SD or median (IQR) according to distribution, and categorical variables as counts and percentages. Differences between patients with and without events were tested with the chi-square (categorical) and Mann–Whitney U test (continuous). The association between plaque burden metrics and regional hMBF was evaluated using Spearman’s rank correlation. Kaplan–Meier survival analysis was conducted to compare event-free survival across per-patient PAV and regional hMBF categories.

Cox proportional hazards regression models were used to estimate hazard ratios (HRs) and 95% confidence intervals (CI) for the association of per-patient PAV or regional hMBF with the composite outcome at 3, 6, and 9 years of follow-up. For each timepoint, datasets were truncated, and events occurring after that time were censored accordingly. Three multivariable Cox regression models were constructed: (i) per-patient PAV, (ii) regional hMBF, and (iii) per-patient PAV and regional hMBF. All multivariable models were adjusted for cardiovascular risk factors (age, sex, hypertension, diabetes) and early revascularization. Covariate selection was informed by clinical variables that were significant predictors of the outcome in univariable Cox analysis. Interaction terms (PAV × Early Revascularization and regional hMBF × Early Revascularization) were entered separately into the combined model (iii) to assess whether early revascularization modified the associations of per-patient PAV or regional hMBF with clinical outcomes throughout the follow-up period.

Robustness was assessed in additional sensitivity analyses: excluding patients who underwent early revascularization, redefining early revascularization with a 3-month threshold, and replacing per-patient PAV with maximum per-vessel PAV or regional hMBF with global hMBF. The two latter substitutions were tested both in the full cohort and after excluding early revascularization. Clinical variables missing at random were imputed using fully conditional specification, generating 20 imputed datasets. A two-sided *p*-value < 0.05 was considered statistically significant. All analyses were conducted using IBM SPSS Statistics version 28 (IBM Corp., Armonk, NY, USA).

## Results

### Baseline characteristics and imaging findings

The study cohort included 1,385 patients with a mean age of 62 ± 9 years, of whom 746 (54%) were male. As shown in Table 1, the prevalence of cardiovascular risk factors was as follows: hypertension in 808 (58%), diabetes mellitus in 251 (18%), and current smoking in 295 (21%) patients. At the time of imaging, 854 (62%) patients were using antiplatelet therapy, 785 (57%) lipid-lowering agents, and 773 (56%) β-blockers. Typical angina was reported by 405 patients (29%). Early revascularization was performed in 281 patients (20%), including 212 percutaneous coronary interventions (PCI) and 69 coronary artery bypass grafting (CABG) procedures, with a median scan-to-procedure time of 49 days (IQR, 18–84).

**Table 1:** Baseline characteristics (*N* = 1385)

Age	62 ± 9 years
Male	*N* = 746 (54%)
BMI	27.6 ± 4.6
Hypertension	*N* = 808 (58%)
Hyperlipidemia	*N* = 792 (57%)
Diabetes Mellitus	*N* = 251 (18%)
Current smoker	*N* = 295 (21%)
Family history of CAD	*N* = 686 (50%)
Typical angina	*N* = 405 (29%)
Medical therapy	
Anti-platelet drugs	*N* = 854 (62%)
Lipid-lowering drugs	*N* = 785 (57%)
β-blockers	*N* = 773 (56%)
ACE-inhibitors	*N* = 278 (22%)
Angiotensin receptor blockers	*N* = 295 (23%)
Calcium channel blockers	*N* = 311 (24%)
Long-acting nitrates	*N* = 92 (13%)
Early Revascularization	*N* = 281 (20%)
Percutaneous coronary intervention	*N* = 212 (75%)
Coronary artery bypass grafting	*N* = 69 (25%)

Demographics, risk factors, medical therapy, and symptoms at the time of scan; early revascularization within 6 months following the scan. Percentages are calculated based on available data; denominators vary due to missing values. Data are mean ± SD or n (%).Abbreviations: *ACE* Angiotensin-converting enzyme; *ARB* Angiotensin receptor blocker; *BMI* Body mass index; *CABG* Coronary artery bypass grafting; *CAD* Coronary artery disease; *PCI* Percutaneous coronary intervention

**Fig. 2:**
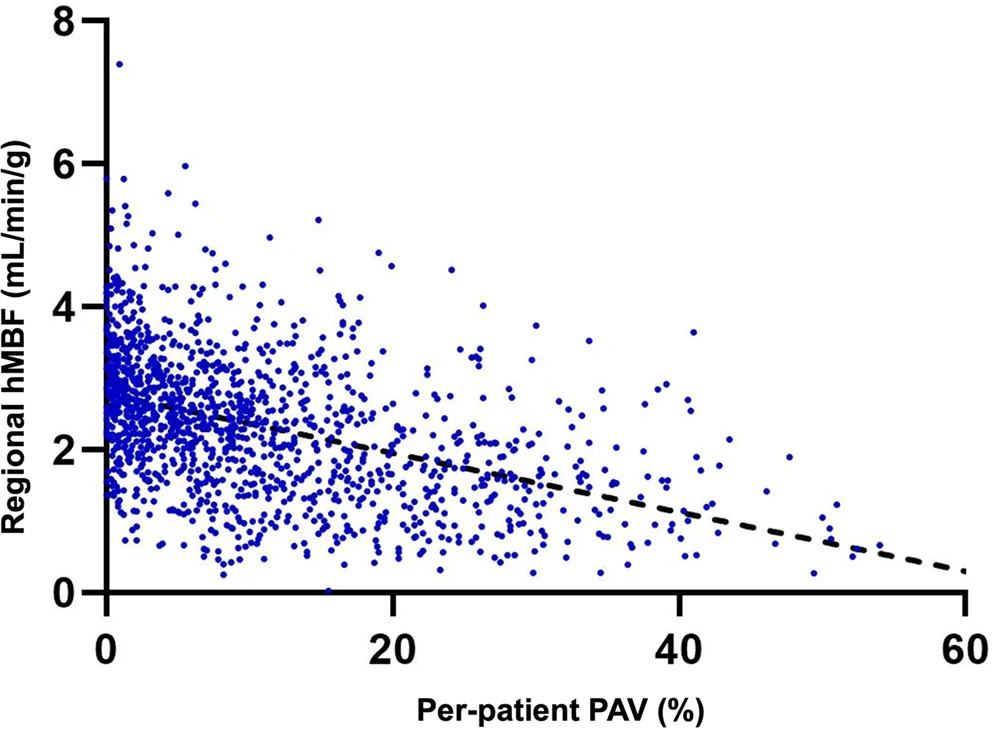
Correlation between Regional hMBF and PAV. Regional hMBF was moderately inversely correlated with PAV (Spearman’s ρ = –0.47; *p* < 0.001), indicating lower perfusion with greater plaque burden. hMBF = hyperemic myocardial blood flow; PAV = percent atheroma volume

As detailed in Table 2, the median per-patient PAV was 7.9% (IQR: 2.7–16.4%). Absence of coronary atherosclerosis (PAV = 0%) was present in 20 patients (1.4%), whereas 384 patients (27.7%) demonstrated extensive plaque burden. The distribution of per-patient maximal diameter stenosis is shown in Table 2. Impaired regional perfusion was detected in 678 patients (49.0%), and was severely reduced in 301 (21.7%). The median regional hMBF across the cohort was 2.33 mL/min/g (1.58–2.95 mL/min/g). Regional hMBF and per-patient PAV demonstrated a moderate inverse correlation (Spearman’s ρ = -0.47, *p* < 0.001) (Fig. 2). Similar inverse correlations were observed between the maximum per-vessel PAV and regional hMBF (ρ = -0.47, *p* < 0.001) and between per-patient PAV and global hMBF (ρ = -0.44, *p* < 0.001).

**Table 2:** CCTA and PET MPI findings according to clinical outcome status

	Total (1385)	No Event (1200)	Event (185)	*p* value
CCTA				
Per-patient PAV (%)	7.9 (2.7–16.4)	7.0 (2.3–14.7)	15.4 (8.1–23.6)	< 0.001
PAV stage				
Stage 0 (PAV 0%)	20 (1.4%)	20 (1.7%)	0 (0%)	< 0.001
Stage 1 (PAV > 0%-5%)	495 (35.7%)	469 (39.1%)	26 (14.1%)	
Stage 2 (PAV > 5%-15%)	486 (35.1%)	419 (34.9%)	67 (36.2%)	
Stage 3 (PAV > 15%)	384 (27.7%)	292 (24.3%)	92 (49.7%)	
Maximum per-vessel PAV	13.4 (4.7–23.7)	12.2 (4.0–21.9)	21.2 (14.1–31.1)	< 0.001
Maximum diameter stenosis (%)	43 (19–65)	38 (16–63)	56 (41–72)	< 0.001
PET MPI				
Regional hMBF (mL/min/g)	2.33 (1.58–2.95)	2.38 (1.66–2.99)	1.85 (1.28–2.58)	< 0.001
Global hMBF (mL/min/g)	3.01 (2.23–3.76)	3.06 (2.31–3.80)	2.62 (1.87–3.49)	< 0.001
Regional Perfusion Categories				
Normal	707 (51%)	640 (53.3%)	67 (36.2%)	< 0.001
Mildly reduced	164 (11.8%)	145 (12.1%)	19 (10.3%)	
Moderately reduced	213 (15.4%)	177 (14.8%)	36 (19.5%)	
Severely reduced	301 (21.7%)	238 (19.8%)	63 (34.1%)	

Values are median (IQR) or n (%). *P* values compare patients with and without the composite outcome; for categorical variables, they represent the overall comparison. Imaging variables are reported at per-patient level. Highest per-vessel PAV was defined as the highest value among LM–LAD, LCx, and RCA. Regional perfusion was categorized as normal (hMBF > 2.3 mL/min/g), mildly reduced (> 2.0–2.3), moderately reduced (> 1.5–2.0), or severely reduced (≤ 1.5 mL/min/g)

*CCTA* Coronary computed tomography angiography; *hMBF* Hyperemic myocardial blood flow; *IQR* Interquartile range; *MPI* Myocardial perfusion imaging; *PAV* Percent atheroma volume; *PET* Positron emission tomography

### Clinical outcomes

Patients were followed for a median duration of 7.1 years (IQR: 4.9–8.6 years, maximum of 13.3 years). During the complete follow-up, the composite outcome occurred in 185 patients (13.4%), comprising 94 all-cause deaths (51%), 59 non-fatal MI (32%), and 32 UA (17%). The observed event rate for composite outcome was 5.2% at 3 years (*n* = 72), 9.5% at 6 years (*n* = 131), and 12.1% at 9 years (*n* = 167).

Patients who experienced events had a significantly higher per-patient PAV compared to those without events (15.4% vs. 7.0%, *p* < 0.001) (Table 2). Similarly, the prevalence of impaired regional perfusion was higher in the event group (63.8% vs. 46.7%, *p* < 0.001). Regional hMBF was significantly lower among patients with events (1.85 vs. 2.38 mL/min/g, *p* < 0.001).

### Association between PAV, hMBF, and clinical outcomes

**Fig. 3:**
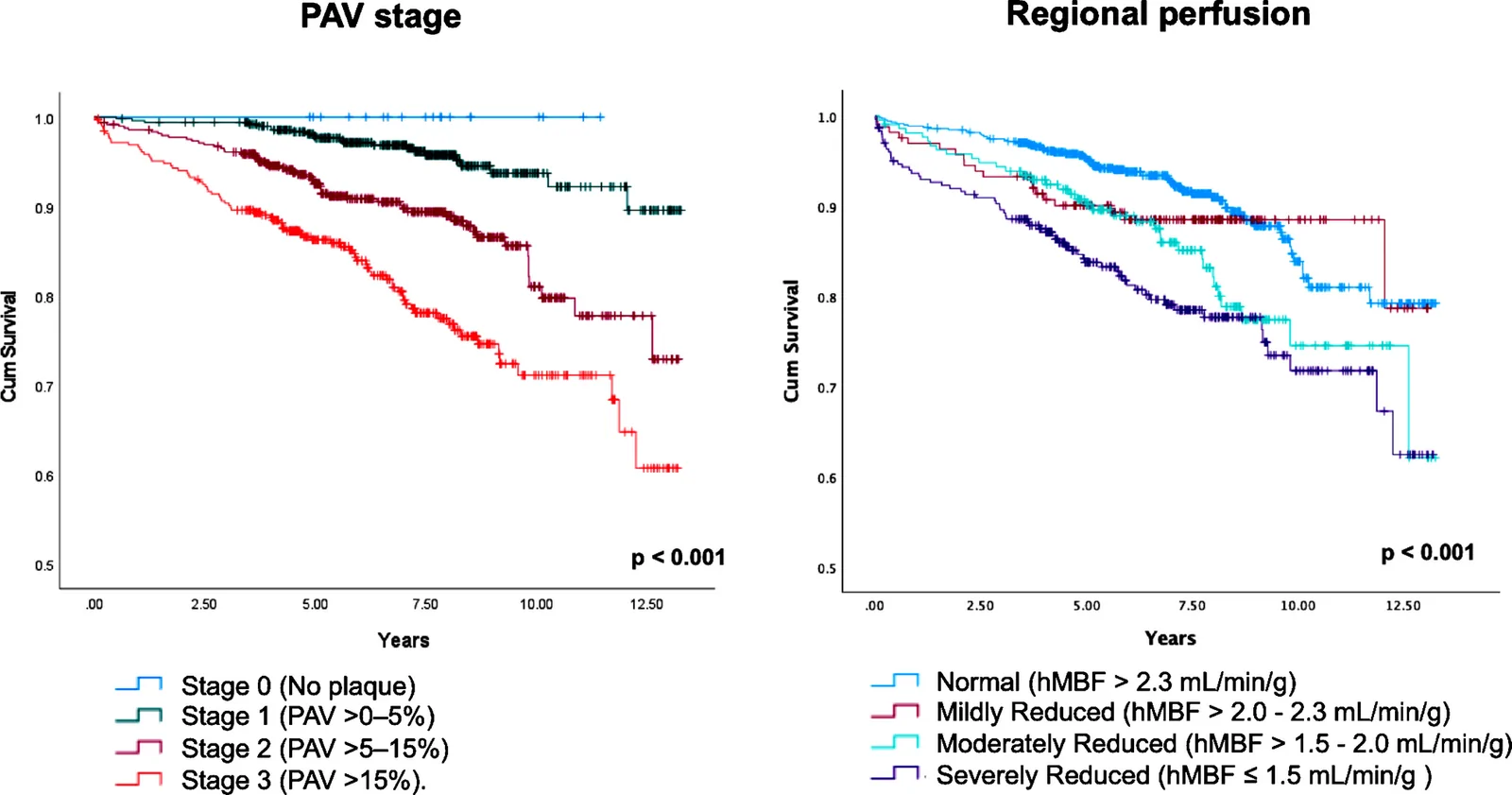
Kaplan–Meier Curves for the Composite Outcome by PAV Stage and Regional Perfusion Category. Kaplan–Meier curves illustrating survival for the composite outcome (all-cause death, myocardial infarction, and unstable angina) by PAV stage and the presence of regional ischemia. PAV was stratified into four stages: Stage 0 (no plaque), Stage 1 (PAV > 0–5%), Stage 2 (PAV > 5–15%), and Stage 3 (PAV > 15%). Regional perfusion was categorized as normal (hMBF > 2.3 mL/min/g), mildly reduced (hMBF > 2.0–2.3 mL/min/g), moderately reduced (hMBF > 1.5–2.0 mL/min/g) or severely reduced (hMBF ≤ 1.5 mL/min/g). PAV = percent atheroma volume; hMBF = hyperemic myocardial blood flow

Kaplan–Meier analysis demonstrated a stepwise decline in the event-free survival with increasing PAV stages and increasing severity of the regional perfusion impairment (Fig. 3). After adjustment for cardiovascular risk factors and early revascularization, per-patient PAV (per 1% increase) was associated with the composite outcome at 3, 6, and 9 years (adjusted hazard ratios [aHR]: 1.055, 1.040, and 1.039, respectively; all *p* < 0.001). Similarly, regional hMBF (per 0.1 mL/min/g decrease) was associated with composite outcomes at 3 years (aHR: 1.071; *p* < 0.001), 6 years (aHR: 1.043; *p* < 0.001), and 9 years (aHR: 1.034; *p* = 0.002), as shown in Supplementary Fig. 1.

**Fig. 4:**
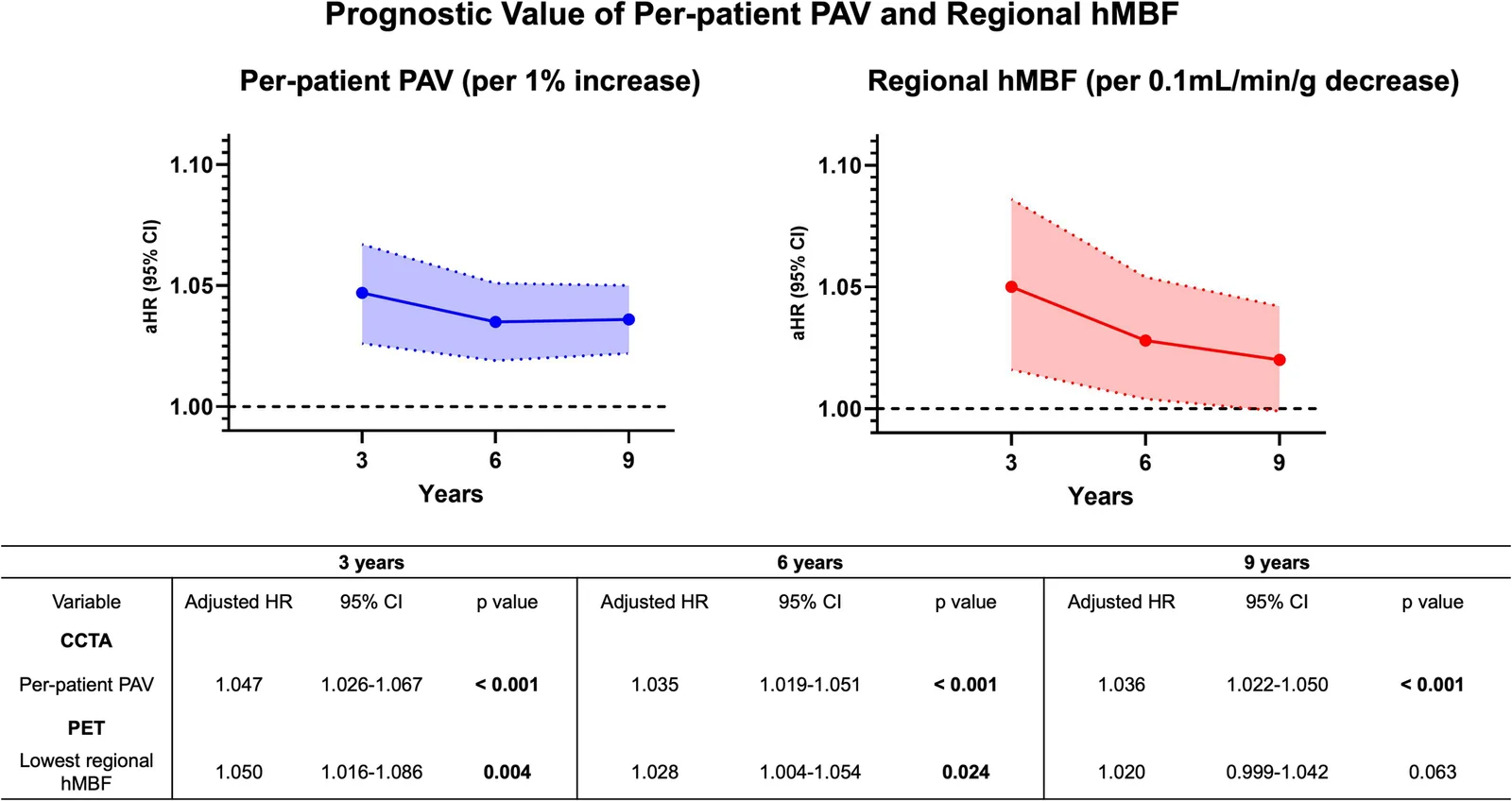
Combined model for PAV and Regional hMBF predicting clinical outcomes at different follow-up times. Adjusted hazard ratios with 95% CI for the composite outcome (all cause death, myocardial infarction and unstable angina) are shown for PAV (per 1% increase, left panel) and regional hMBF (per 0.1 mL/min/g decrease, right panel) at 3-, 6-, and 9-year follow-up timepoints. Both imaging variables were included in the same multivariable Cox regression model, adjusted for cardiovascular risk factors (CVRF: age, sex, hypertension, and diabetes) and early revascularization. aHR = adjusted hazard ratio; CI = confidence interval; CVRF = cardiovascular risk factors; PAV = percent atheroma volume; hMBF = hyperemic myocardial blood flow

In a combined model including both per-patient PAV and regional hMBF (Fig. 4), adjusted for cardiovascular risk factors and early revascularization, per-patient PAV remained a consistent predictor of the outcome at 3, 6, and 9 years (aHR: 1.047, 1.035, and 1.036, respectively; all *p* < 0.001). Regional hMBF was independently associated with events at 3 years (aHR: 1.050; *p* = 0.004) and 6 years (aHR: 1.028; *p* = 0.024), but not at 9 years (aHR: 1.020; *p* = 0.063).

**Fig. 5:**
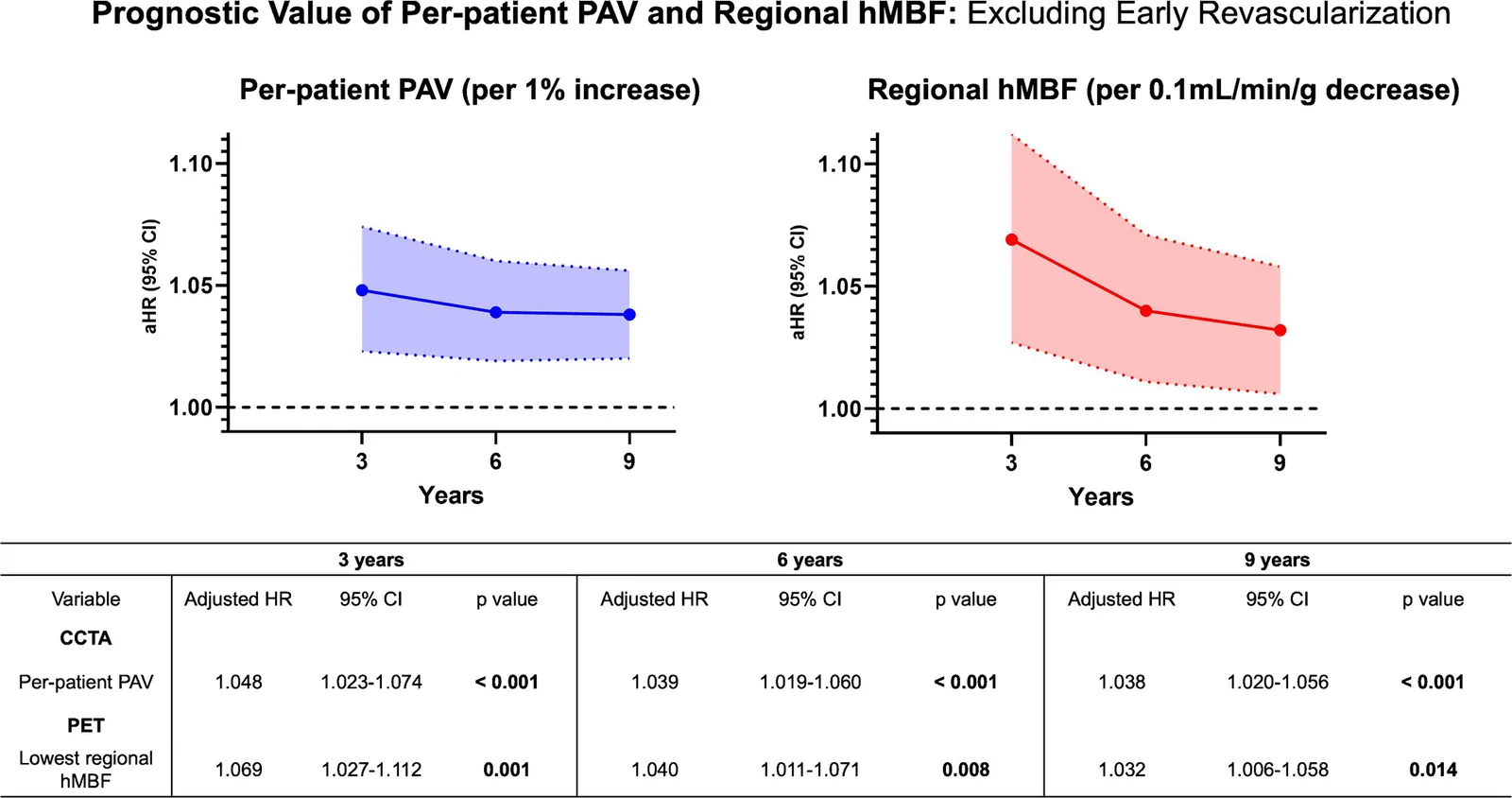
Combined Model for PAV and Regional hMBF predicting clinical outcomes in patients without early revascularization (*n* = 1,104), at different follow-up times. Adjusted hazard ratios with 95% CI for the composite outcome (all-cause death, myocardial infarction, and unstable angina) are shown for PAV (per 1% increase, left panel) and regional hMBF (per 0.1 mL/min/g decrease, right panel) at 3-, 6-, and 9-year follow-up timepoints. Patients who underwent early revascularization were excluded, yielding a final cohort of 1,104 individuals. Both imaging variables were included in the same multivariable Cox regression model, adjusted for cardiovascular risk factors (CVRF: age, sex, hypertension, and diabetes). aHR = adjusted hazard ratio; CI = confidence interval; CVRF = cardiovascular risk factors; PAV = percent atheroma volume; hMBF = hyperemic myocardial blood flow

Additionally, interaction terms between early revascularization and per-patient PAV, as well as between early revascularization and regional hMBF, were statistically significant (*p* = 0.035 and *p* = 0.025, respectively), suggesting that the prognostic impact of plaque burden and regional hMBF may be modified by revascularization. To account for this, a sensitivity analysis excluding patients who underwent early revascularization was performed. In this cohort (*n* = 1,104), both per-patient PAV and regional hMBF were significantly associated with the outcome across all timepoints, as shown in Fig. 5.

Using alternative plaque burden and perfusion metrics (Supplementary Table 1), two combined models were evaluated. In the model of maximum per-vessel PAV and regional hMBF, per-vessel PAV was associated with the composite outcome at 3, 6, and 9 years, whereas regional hMBF was associated at 3 and 6 years but not at 9 years (aHR 1.021, 95% CI 1.000–1.043; *p* = 0.050); after excluding patients who underwent early revascularization, per-vessel PAV remained associated at all timepoints and regional hMBF became associated at 9 years as well (aHR 1.030, 95% CI 1.007–1.054; *p* = 0.012). In the model of per-patient PAV and global hMBF, per-patient PAV was associated at all timepoints and global hMBF only at 3 years, unchanged after excluding early revascularization.

When early revascularization was defined by a 3-month threshold (Supplementary Table 2), per-patient and maximum per-vessel PAV were associated at 3, 6, and 9 years, both in the full cohort and after excluding early revascularization, whereas regional hMBF was associated at 3 and 6 years but not at 9 years. Global hMBF was associated only at 3 years, in the full cohort (aHR 1.028, 95% CI 1.001–1.056; *p* = 0.045).

## Discussion

In this large, two-center cohort, coronary atherosclerotic plaque burden assessed by CCTA and myocardial ischemia measured by [^15^O]H₂O PET were found to be complementary, time-dependent predictors of future adverse events. First, when the prognostic value of per-patient PAV and regional hMBF was studied separately, both measures were independently associated with clinical outcomes in short- to long-term follow-up. When they were combined into the multivariable model, per-patient PAV remained a strong predictor of events over time, while the prognostic value of regional hMBF diminished and was no longer statistically significant at nine years of follow-up. Second, after excluding patients who underwent early revascularization, both metrics were independently associated with adverse outcomes throughout the follow-up period. Third, while the strength of association between regional hMBF and clinical events appeared to attenuate over time, the relationship between plaque burden and outcomes remained relatively stable.

Myocardial ischemia is inherently linked to the extent and distribution of coronary atherosclerosis. Its development is influenced not only by the presence of plaque, but also by its focal severity, the diffuseness of vascular involvement, and the functional integrity of the coronary microvasculature [22]. The interplay among these factors determines the extent of myocardial perfusion impairment. Therefore, ischemia represents the functional consequence of the underlying atherosclerotic process.

The prognostic role of myocardial ischemia and its value in guiding therapeutic decision-making has become increasingly debated. This skepticism arises, in part, from randomized trials showing that ischemia-guided revascularization, although effective in relieving ischemia and symptoms, does not significantly improve clinical outcomes compared to guideline-directed medical therapy in patients with chronic CAD [23, 24]. However, these trials primarily employed earlier-generation MPI techniques or even non-imaging exercise testing, which may have failed to fully characterize the burden and complexity of myocardial ischemia. In contrast, advanced modalities such as quantitative PET MPI offer a more precise, continuous evaluation of myocardial perfusion and ischemic severity, rather than relying on binary thresholds [25]. Indeed, observational studies have consistently shown that PET MPI provides incremental prognostic value beyond the anatomical information offered by CCTA, reinforcing the clinical relevance of ischemia for risk stratification [8, 26, 27]. This study is among the first to demonstrate that myocardial ischemia, as assessed by PET MPI, is independently associated with clinical outcomes when integrated with AI-based quantitative CCTA, a technique that is rapidly emerging as a tool for cardiovascular risk assessment by providing new insights into coronary biology.

Consistent with our findings, a prior study utilizing machine learning models in a partly overlapping cohort with the present study suggested that the prognostic value of myocardial ischemia, beyond anatomical metrics and without inclusion of plaque quantification, was most evident in predicting short-term events [26]. Similarly, CCTA studies examining culprit lesion characteristics have shown that, compared with later acute coronary syndromes, early events are more often associated with more severe diameter stenosis and high-risk plaque features, both linked to ischemia-producing lesions [28, 29]. Patients with ischemia-driving lesions, identified through invasive assessment and managed with medical therapy, appear more likely to experience adverse events or require urgent revascularization within the first one to two years of follow-up, as observed in the FAME 2 trial and supported by prospective registries such as IRIS-FFR [30, 31]. These observations suggest that regional hMBF, as a marker of focal flow impairment, is more closely associated with short- and intermediate-term risk. In contrast, total atherosclerotic burden appears to provide a more stable prognostic value extending into the long term.

Notably, after excluding patients who underwent early revascularization and therefore potential ischemia relief, the association between regional hMBF and the composite outcome strengthened numerically across all follow-up timepoints, most prominently in the short term, and reached statistical significance also at nine years. Although these results should be interpreted with caution due to potential residual confounding (i.e., selection of patients for early revascularization), they underscore the impact of revascularization on the prognostic utility of hMBF, and suggest that untreated ischemia retains independent prognostic value beyond anatomical imaging, even during long-term follow-up. These findings were consistent when early revascularization was defined using a 3-month threshold and when alternative plaque burden and perfusion metrics were applied, supporting the robustness of the observed time-dependent associations.

The clinical implications of these findings should be considered in light of current evidence. Recent scientific statements support AI-based quantitative plaque analysis to enhance risk assessment and help guide preventive therapy in patients with plaque on CCTA [32, 33]. PAV may further improve long-term risk stratification and support more intensive prevention, particularly in non-obstructive CAD, where anatomic risk is otherwise underestimated. Regional hMBF identifies impaired myocardial perfusion and higher near-term risk, and may inform ischemia characterization, symptom-directed management, and closer short-term follow-up. Staging frameworks linking plaque volume to preventive targets have recently been proposed, although optimal thresholds are still being defined [32, 33]. Further prospective data are awaited to define the benefit of plaque-guided interventions and refine their role in clinical practice.

## Study limitations

Some limitations of this study should be considered. First, we did not have comprehensive data on patient’s medical therapy following imaging. Including information on pharmacologic treatment might have strengthened the observed associations between imaging metrics and outcomes. Second, the retrospective design limited adjudication of more specific outcomes such as cardiovascular mortality; we therefore used all-cause mortality; non-fatal MI and unstable angina were investigator-verified by standardized ascertainment. Third, scans were acquired using different hardware, though all met the technical standards recommended by the Society of Cardiovascular Computed Tomography, and repeated follow-up scans were not performed due to real-world observational study setting [34]. Fourth, our findings pertain specifically to absolute regional hMBF from [^15^O]H₂O PET and may not extend to reserve-based indices (myocardial flow reserve, coronary flow capacity) or other tracers (e.g., ^82^Rb, ^13^N-ammonia), for which reserve-based metrics have shown comparable or superior long-term prognostic value [25, 35, 36]. Fifth, imaging protocols varied between the institutions: patients in Amsterdam, the Netherlands, underwent a classical hybrid imaging protocol including both CCTA and PET MPI routinely, whereas patients in Turku, Finland, selectively underwent PET MPI in case of suspected obstructive CCTA findings, in agreement with the current guideline recommendations [8, 37]. As both represent accepted clinical strategies, the inclusion of patients imaged under either approach strengthens rather than limits the generalizability of our findings across the spectrum of CAD. Finally, potential residual confounding is an inherent limitation of any retrospective observational study.

## Conclusion

In patients with suspected CAD, coronary atherosclerotic plaque burden assessed by CCTA and myocardial ischemia evaluated by [^15^O]H₂O PET were found to be independent predictors of future adverse events. Plaque burden was consistently associated with adverse clinical outcomes over time, serving as a stable risk marker throughout the long-term 9-year follow-up. Regional hMBF, reflecting the severity of myocardial ischemia, was associated with short- and intermediate-term adverse events, likely influenced by the effects of early revascularization. These findings support the complementary role of anatomical and functional imaging modalities for individualized risk assessment and management in chronic CAD.

### Clinical perspectives

## Competency in medical knowledge

Quantitative assessment of coronary plaque burden and myocardial perfusion using combined CCTA and [^15^O]H₂O PET provides complementary information on structural and functional disease severity in suspected CAD patients. Plaque burden was a consistent determinant of outcomes throughout follow-up, whereas regional hMBF related primarily to early and intermediate events.

## Translational outlook

Quantitative plaque and perfusion imaging may reflect separate but interrelated aspects of coronary pathology. Their integration might allow a better understanding of the mechanisms driving CAD progression and patient prognosis.

## Supplementary Information

Below is the link to the electronic supplementary material.

Supplementary file1 (DOCX 270 KB)

## Data Availability

Study data are not publicly available due to privacy and ethical restrictions.

## Abbreviations

AI: Artificial Intelligence
CAD: Coronary Artery Disease
CCTA: Coronary Computed Tomography Angiography
hMBF: Hyperemic Myocardial Blood Flow
MPI: Myocardial Perfusion Imaging
PAV: Percent Atheroma Volume
PET: Positron Emission Tomography

## References

[1] Mancini GBJ, Hartigan PM, Shaw LJ, et al. Predicting outcome in the COURAGE Trial (Clinical Outcomes Utilizing Revascularization and Aggressive Drug Evaluation): Coronary Anatomy Versus Ischemia. JACC Cardiovasc Interv. 2014;7:195–201. 10.1016/j.jcin.2013.10.017 24440015

[2] Reynolds HR, Shaw LJ, Min JK, et al. Outcomes in the ISCHEMIA trial based on coronary artery disease and ischemia severity. Circulation. 2021;144:1024–38. 10.1161/CIRCULATIONAHA.120.049755 PMC8478888 34496632

[3] Patel KK, Peri-Okonny PA, Giorgetti A, Shaw LJ, Gimelli A. Value of ischemia and coronary anatomy in prognosis and guiding revascularization among patients with stable ischemic heart disease. Circ Cardiovasc Imaging. 2024;17:e016587. 10.1161/CIRCIMAGING.123.016587 PMC11439561 39247957

[4] Rozanski A, Miller RJH, Gransar H, et al. Benefit of early revascularization based on inducible ischemia and left ventricular ejection fraction. J Am Coll Cardiol. 2022;80:202–15. 10.1016/j.jacc.2022.04.052 35835493

[5] Dzaye O, Razavi AC, Blaha MJ, Mortensen MB. Evaluation of coronary stenosis versus plaque burden for atherosclerotic cardiovascular disease risk assessment and management. Curr Opin Cardiol. 2021;36:769–75. 10.1097/HCO.0000000000000911 PMC8547346 34620792

[6] Dahdal J, Jukema RA, Maaniitty T, et al. CCTA-derived coronary plaque burden offers enhanced prognostic value over CAC scoring in suspected CAD patients. Eur Heart J Cardiovasc Imaging. 2025;26:945–54. 10.1093/ehjci/jeaf093 40131307

[7] Nurmohamed NS, Bom MJ, Jukema RA, et al. AI-guided quantitative plaque staging predicts long-term cardiovascular outcomes in patients at risk for atherosclerotic CVD. JACC Cardiovasc Imaging. 2024;17:269–80. 10.1016/j.jcmg.2023.05.020 37480907

[8] Maaniitty T, Stenström I, Bax JJ, et al. Prognostic value of coronary CT angiography with selective PET perfusion imaging in coronary artery disease. JACC Cardiovasc Imaging. 2017;10:1361–70. 10.1016/j.jcmg.2016.10.025 28528146

[9] Driessen RS, Bom MJ, van Diemen PA, et al. Incremental prognostic value of hybrid [15O]H2O positron emission tomography-computed tomography: combining myocardial blood flow, coronary stenosis severity, and high-risk plaque morphology. Eur Heart J Cardiovasc Imaging. 2020;21:1105–13. 10.1093/ehjci/jeaa192 PMC7971168 32959061

[10] van Diemen PA, Bom MJ, Driessen RS, et al. Prognostic value of RCA pericoronary adipose tissue CT-attenuation beyond high-risk plaques, plaque volume, and ischemia. JACC Cardiovasc Imaging. 2021;14:1598–610. 10.1016/j.jcmg.2021.02.026 33958312

[11] Choi AD, Marques H, Kumar V, et al. CT evaluation by artificial intelligence for atherosclerosis, stenosis and vascular morphology (CLARIFY): a multi-center, international study. J Cardiovasc Comput Tomogr. 2021;15:470–6. 10.1016/j.jcct.2021.05.004 34127407

[12] Jukema RA, Maaniitty T, Nurmohamed NS, et al. Location-specific prognostic significance of plaque burden, stenosis, and plaque morphology in coronary artery disease. Eur Heart J Cardiovasc Imaging. 2025;26:22–9. 10.1093/ehjci/jeae214 39163147

[13] Min JK, Chang H-J, Andreini D, et al. Coronary CTA plaque volume severity stages according to invasive coronary angiography and FFR. J Cardiovasc Comput Tomogr. 2022;16:415–22. 10.1016/j.jcct.2022.03.001 35379596

[14] Cerqueira MD, Weissman NJ, Dilsizian V, et al. Standardized myocardial segmentation and nomenclature for tomographic imaging of the heart. A statement for healthcare professionals from the Cardiac Imaging Committee of the Council on Clinical Cardiology of the American Heart Association. Circulation. 2002;105:539–42. 10.1161/hc0402.102975 11815441

[15] Harms HJ, Knaapen P, de Haan S, Halbmeijer R, Lammertsma AA, Lubberink M. Automatic generation of absolute myocardial blood flow images using [15O]H_2_O and a clinical PET/CT scanner. Eur J Nucl Med Mol Imaging. 2011;38:930–9. 10.1007/s00259-011-1730-3 PMC3070081 21271246

[16] Harms HJ, Nesterov SV, Han C, et al. Comparison of clinical non-commercial tools for automated quantification of myocardial blood flow using oxygen-15-labelled water PET/CT. Eur Heart J Cardiovasc Imaging. 2014;15:431–41. 10.1093/ehjci/jet177 24107905

[17] Bom MJ, van Diemen PA, Driessen RS, et al. Prognostic value of [15O]H_2_O positron emission tomography-derived global and regional myocardial perfusion. Eur Heart J Cardiovasc Imaging. 2020;21:777–86. 10.1093/ehjci/jez258 31620792

[18] Danad I, Uusitalo V, Kero T, et al. Quantitative assessment of myocardial perfusion in the detection of significant coronary artery disease: cutoff values and diagnostic accuracy of quantitative [(^15^)O]H_2_O PET imaging. J Am Coll Cardiol. 2014;64:1464–75. 10.1016/j.jacc.2014.05.069 25277618

[19] Collet JP, Thiele H, Barbato E, et al. 2020 ESC guidelines for the management of acute coronary syndromes in patients presenting without persistent ST-segment elevation. Eur Heart J. 2021;42:1289–367. 10.1093/eurheartj/ehaa575 32860058

[20] Bär S, Knuuti J, Saraste A, et al. Outcomes with revascularization vs. medical therapy according to plaque burden from coronary computed tomography angiography. Eur Heart J Cardiovasc Imaging. 2026;27:554–66. 10.1093/ehjci/jeaf372 41460775

[21] Jukema RA, Maaniitty T, Nurmohamed NS, et al. The time-varying prognostic value of stenosis and plaque burden in coronary artery disease. Eur Heart J Cardiovasc Imaging. 2026;27:696–703. 10.1093/ehjci/jeag022 PMC13021277 41604434

[22] Hoshino M, Jukema RA, Hoek R, et al. Microvascular resistance reserve in relation to total and vessel-specific atherosclerotic burden. Eur Heart J Cardiovasc Imaging. 2024;26:481–8. 10.1093/ehjci/jeae293 PMC11879247 39531645

[23] Maron DJ, Hochman JS, Reynolds HR, et al. Initial invasive or conservative strategy for stable coronary disease. N Engl J Med. 2020;382:1395–407. 10.1056/NEJMoa1915922 PMC7263833 32227755

[24] Boden WE, O’Rourke RA, Teo KK, et al. Optimal medical therapy with or without PCI for stable coronary disease. N Engl J Med. 2007;356:1503–16. 10.1056/NEJMoa070829 17387127

[25] Dahdal J, Jukema RA, Harms HJ, et al. PET myocardial perfusion imaging: trends, challenges, and opportunities. J Nucl Cardiol. 2024;40:102011. 10.1016/j.nuclcard.2024.102011 39067504

[26] Lehtonen E, Kujala I, Tamminen J, et al. Incremental prognostic value of downstream positron emission tomography perfusion imaging after coronary computed tomography angiography: a study using machine learning. Eur Heart J Cardiovasc Imaging. 2024;25:285–92. 10.1093/ehjci/jead246 PMC10824480 37774503

[27] Jukema R, Maaniitty T, van Diemen P, et al. Warranty period of coronary computed tomography angiography and [15O]H2O positron emission tomography in symptomatic patients. Eur Heart J Cardiovasc Imaging. 2023;24:304–11. 10.1093/ehjci/jeac258 36585755

[28] Driessen RS, Stuijfzand WJ, Raijmakers PG, et al. Effect of plaque burden and morphology on myocardial blood flow and fractional flow reserve. J Am Coll Cardiol. 2018;71:499–509. 10.1016/j.jacc.2017.11.054 29406855

[29] van den Hoogen IJ, Stuijfzand WJ, Gianni U, et al. Early versus late acute coronary syndrome risk patterns of coronary atherosclerotic plaque. Eur Heart J Cardiovasc Imaging. 2022;23:1314–23. 10.1093/ehjci/jeac114 35904766

[30] Xaplanteris P, Fournier S, Pijls NHJ, et al. Five-year outcomes with PCI guided by fractional flow reserve. N Engl J Med. 2018;379:250–9. 10.1056/NEJMoa1803538 29785878

[31] Ahn J-M, Park D-W, Shin E-S, et al. Fractional flow reserve and cardiac events in coronary artery disease. Circulation. 2017;135:2241–51. 10.1161/CIRCULATIONAHA.116.024433 28356440

[32] Chandrashekhar Y, Blankstein R, Shaw LJ, et al. Quantitative coronary plaque analysis in clinical practice: 2025 ACC Scientific Statement. JACC Cardiovasc Imaging. 2025;19:637–52. 10.1016/j.jcmg.2025.11.008 41405512

[33] Slipczuk L, Blankstein R, Bucciarelli-Ducci C, et al. State of the art: evaluation and medical management of nonobstructive coronary artery disease in patients with chest pain: a scientific statement from the American Heart Association. Circulation. 2025;152:e443–66. 10.1161/CIR.0000000000001394 41208705

[34] Abbara S, Blanke P, Maroules CD, et al. SCCT guidelines for the performance and acquisition of coronary computed tomographic angiography: a report of the Society of Cardiovascular Computed Tomography Guidelines Committee: endorsed by the North American Society for Cardiovascular Imaging (NASCI). J Cardiovasc Comput Tomogr. 2016;10:435–49. 10.1016/j.jcct.2016.10.002 27780758

[35] Khan MS, Bateman TM, Case JA, Sperry BW. Beyond flow reserve: comparative prognostic value of myocardial blood flow reserve, peak stress myocardial blood flow, and blood flow reserve corrected for resting rate pressure product in patients undergoing positron emission tomography myocardial perfusion imaging. J Nucl Cardiol. 2026;55:102540. 10.1016/j.nuclcard.2025.102540 41205795

[36] Kelshiker MA, Seligman H, Howard JP, et al. Coronary flow reserve and cardiovascular outcomes: a systematic review and meta-analysis. Eur Heart J. 2022;43:1582–93. 10.1093/eurheartj/ehab775 PMC9020988 34849697

[37] Vrints C, Andreotti F, Koskinas KC, et al. 2024 ESC guidelines for the management of chronic coronary syndromes: developed by the task force for the management of chronic coronary syndromes of the European Society of Cardiology (ESC) endorsed by the European Association for Cardio-Thoracic Surgery (EACTS). Eur Heart J. 2024;45:3415–537. 10.1093/eurheartj/ehae177 39210710

