# Supplementary material for: Time-dependent prognostic value of coronary plaque burden and hyperemic myocardial blood flow
Source: Eur J Nucl Med Mol Imaging. 2026 Aug 30;53(13):8023–34. doi: 10.1007/s00259-026-08135-3 (PMC13727843; doi:10.1007/s00259-026-08135-3)
Supplement: Supplementary file 1 [file 259_2026_8135_MOESM1_ESM.docx]

**Supplementary Figure 1. Separate Cox Models of PAV and hMBF Across Follow-up.**

*Legend Supplementary Figure 1.*

Adjusted hazard ratios with 95% confidence intervals for the composite outcome (all cause death, myocardial infarction and unstable angina) are shown for PAV (per 1% increase, left) and regional hMBF (per 0.1 mL/min/g decrease, right) at 3-, 6-, and 9-year follow-up timepoints. Imaging variables (PAV and regional hMBF) were evaluated in separate multivariable Cox regression models adjusted for cardiovascular risk factors (CVRF: age, sex, hypertension, and diabetes) and early revascularization. aHR = adjusted hazard ratio; CI = confidence interval; CVRF = cardiovascular risk factors; PAV = percent atheroma volume; hMBF = hyperemic myocardial blood flow.

**Supplementary Table 1. Combined Models Using Alternative Plaque and Perfusion Metrics Across Follow-up.**

| **Model / Variable** | **3-year aHR (95% CI)** | **p** | **6-year aHR (95% CI)** | **p** | **9-year aHR (95% CI)** | **p** |
| --- | --- | --- | --- | --- | --- | --- |
| **Full cohort (N=1,385)** | | | | | | |
| *Max per-vessel PAV + regional hMBF* | | | | | | |
| Max per-vessel PAV | 1.033 (1.016–1.051) | <0.001 | 1.025 (1.011–1.039) | <0.001 | 1.026 (1.014–1.038) | <0.001 |
| Regional hMBF | 1.052 (1.017–1.088) | 0.003 | 1.030 (1.005–1.055) | 0.018 | 1.021 (1.000–1.043) | 0.050 |
| *Per-patient PAV + global hMBF* | | | | | | |
| Per-patient PAV | 1.050 (1.030–1.070) | <0.001 | 1.038 (1.022–1.054) | <0.001 | 1.038 (1.024–1.053) | <0.001 |
| Global hMBF | 1.033 (1.004–1.062) | 0.026 | 1.013 (0.993–1.034) | 0.211 | 1.007 (0.989–1.024) | 0.461 |
| **Excluding early revascularization (N=1,104)** | | | | | | |
| *Max per-vessel PAV + regional hMBF* | | | | | | |
| Max per-vessel PAV | 1.040 (1.019–1.061) | <0.001 | 1.030 (1.013–1.047) | <0.001 | 1.029 (1.015–1.044) | <0.001 |
| Regional hMBF | 1.061 (1.022–1.100) | 0.002 | 1.037 (1.010–1.066) | 0.008 | 1.030 (1.007–1.054) | 0.012 |
| *Per-patient PAV + global hMBF* | | | | | | |
| Per-patient PAV | 1.055 (1.032–1.079) | <0.001 | 1.043 (1.025–1.062) | <0.001 | 1.043 (1.027–1.060) | <0.001 |
| Global hMBF | 1.036 (1.004–1.069) | 0.027 | 1.020 (0.997–1.044) | 0.084 | 1.013 (0.994–1.033) | 0.179 |

*Legend Supplementary Table 1.*

*Adjusted hazard ratios with 95% CI for the composite outcome (all-cause death, myocardial infarction, and unstable angina) at 3-, 6-, and 9-year follow-up timepoints, using alternative plaque burden and perfusion metrics. Two combined multivariable Cox regression models are shown: maximum per-vessel PAV with regional hMBF, and per-patient PAV with global hMBF. PAV is expressed per 1% increase and hMBF per 0.1 mL/min/g decrease. Results are presented for the full cohort (n = 1,385) and after excluding patients who underwent early revascularization (n = 1,104). Models were adjusted for cardiovascular risk factors (CVRF: age, sex, hypertension, and diabetes) and early revascularization; the models excluding early revascularization were adjusted for CVRF only. aHR = adjusted hazard ratio; CI = confidence interval; CCTA = coronary computed tomography angiography; PET = positron emission tomography; PAV = percent atheroma volume; hMBF = hyperemic myocardial blood flow.*

**Supplementary Table 2. Combined Models Using a 3-Month Early Revascularization Threshold Across Follow-up.**

| **Model / Variable** | **3-year aHR (95% CI)** | | **p** | **6-year aHR (95% CI)** | **p** | **9-year aHR (95% CI)** | **p** |
| --- | --- | --- | --- | --- | --- | --- | --- |
| **Full cohort (N=1,385)** | | | | | | | |
| *Per-patient PAV + regional hMBF* | | | | | | | |
| Per-patient PAV | 1.046 (1.026–1.067) | <0.001 | | 1.034 (1.018–1.050) | <0.001 | 1.035 (1.020–1.049) | <0.001 |
| Regional hMBF | 1.042 (1.010–1.076) | 0.011 | | 1.025 (1.001–1.049) | 0.039 | 1.015 (0.995–1.036) | 0.139 |
| *Max per-vessel PAV + regional hMBF* | | | | | | | |
| Max per-vessel PAV | 1.032 (1.015–1.050) | <0.001 | | 1.024 (1.010–1.038) | <0.001 | 1.025 (1.013–1.037) | <0.001 |
| Regional hMBF | 1.046 (1.012–1.080) | 0.007 | | 1.027 (1.003–1.051) | 0.028 | 1.017 (0.996–1.038) | 0.107 |
| *Per-patient PAV + global hMBF* | | | | | | | |
| Per-patient PAV | 1.048 (1.028–1.069) | <0.001 | | 1.037 (1.021–1.053) | <0.001 | 1.037 (1.023–1.051) | <0.001 |
| Global hMBF | 1.028 (1.001–1.056) | 0.045 | | 1.012 (0.992–1.031) | 0.250 | 1.004 (0.987–1.021) | 0.638 |
| **Excluding early revascularization within 3 months (N=1,163)** | | | | | | | |
| *Per-patient PAV + regional hMBF* | | | | | | | |
| Per-patient PAV | 1.049 (1.025–1.073) | <0.001 | | 1.040 (1.022–1.059) | <0.001 | 1.040 (1.024–1.057) | <0.001 |
| Regional hMBF | 1.050 (1.015–1.087) | 0.005 | | 1.026 (1.000–1.052) | 0.047 | 1.019 (0.997–1.041) | 0.094 |
| *Max per-vessel PAV + regional hMBF* | | | | | | | |
| Max per-vessel PAV | 1.038 (1.017–1.059) | <0.001 | | 1.030 (1.014–1.046) | <0.001 | 1.029 (1.015–1.044) | <0.001 |
| Regional hMBF | 1.053 (1.017–1.090) | 0.003 | | 1.028 (1.002–1.054) | 0.033 | 1.021 (0.999–1.043) | 0.067 |
| Per-patient PAV + global hMBF | | | | | | | |
| Per-patient PAV | 1.053 (1.030–1.076) | <0.001 | | 1.043 (1.025–1.061) | <0.001 | 1.043 (1.027–1.059) | <0.001 |
| Global hMBF | 1.029 (1.000–1.059) | 0.050 | | 1.013 (0.992–1.034) | 0.232 | 1.006 (0.988–1.025) | 0.498 |

*Legend Supplementary Table 2.*

*Adjusted hazard ratios with 95% CI for the composite outcome (all-cause death, myocardial infarction, and unstable angina) at 3-, 6-, and 9-year follow-up timepoints, with early revascularization redefined as PCI or CABG within 3 months of the index CCTA. Three combined multivariable Cox regression models are shown: per-patient PAV with regional hMBF, maximum per-vessel PAV with regional hMBF, and per-patient PAV with global hMBF. PAV is expressed per 1% increase and hMBF per 0.1 mL/min/g decrease. Results are presented for the full cohort (n = 1,385) and after excluding patients who underwent early revascularization within 3 months (n = 1,163). Models were adjusted for cardiovascular risk factors (CVRF: age, sex, hypertension, and diabetes) and early revascularization; the models excluding early revascularization were adjusted for CVRF only. aHR = adjusted hazard ratio; CI = confidence interval; CABG = coronary artery bypass grafting; CCTA = coronary computed tomography angiography; PCI = percutaneous coronary intervention; PET = positron emission tomography; PAV = percent atheroma volume; hMBF = hyperemic myocardial blood flow.*
